# Identification of *Cryptosporidium* Species in Fish from Lake Geneva (Lac Léman) in France

**DOI:** 10.1371/journal.pone.0133047

**Published:** 2015-07-27

**Authors:** Gabriela Certad, Jean Dupouy-Camet, Nausicaa Gantois, Ourida Hammouma-Ghelboun, Muriel Pottier, Karine Guyot, Sadia Benamrouz, Marwan Osman, Baptiste Delaire, Colette Creusy, Eric Viscogliosi, Eduardo Dei-Cas, Cecile Marie Aliouat-Denis, Jérôme Follet

**Affiliations:** 1 Biologie et Diversité des Pathogènes Eucaryotes Emergents (BDEEP), Centre d'Infection et d'Immunité de Lille (CIIL), Institut Pasteur de Lille, INSERM U1019, CNRS UMR 8402, Université de Lille, Lille, France; 2 Université Paris Descartes, Assistance Publique Hôpitaux de Paris, Parasitologie-Mycologie, Hôpital Cochin, Paris, France; 3 Laboratoire de Biotechnologie et Gestion des Agents Pathogènes en Agriculture, Institut Supérieur d’Agriculture de Lille, Lille, France; 4 Faculté de Pharmacie, Université de Lille, Lille, France; 5 Ecologie et Biodiversité, Faculté Libre des Sciences et Technologies de Lille, Université Catholique de Lille, Lille, France; 6 Laboratoire Microbiologie, Santé et Environnement, Centre AZM pour la Recherche en Biotechnologie et ses Applications, Université Libanaise, Tripoli, Lebanon; 7 Service d’Anatomie et de Cytologie Pathologiques, Groupe Hospitalier de l’Université Catholique de Lille, Lille, France; 8 Centre Hospitalier Régional et Universitaire de Lille, Université Lille Nord de France, Lille, France; 9 Laboratoire BioMEMS, Univ.Lille, CNRS, ISEN, Univ.Valenciennes, UMR 8520, IEMN, Institut d'Electronique de Microélectronique et de Nanotechnologie, F 59 000, Lille, France; Institut national de la santé et de la recherche médicale - Institut Cochin, FRANCE

## Abstract

*Cryptosporidium*, a protozoan parasite that can cause severe diarrhea in a wide range of vertebrates including humans, is increasingly recognized as a parasite of a diverse range of wildlife species. However, little data are available regarding the identification of *Cryptosporidium* species and genotypes in wild aquatic environments, and more particularly in edible freshwater fish. To evaluate the prevalence of *Cryptosporidium*spp. in fish from Lake Geneva (Lac Léman) in France, 41 entire fish and 100 fillets (cuts of fish flesh) were collected from fishery suppliers around the lake. Nested PCR using degenerate primers followed by sequence analysis was used. Five fish species were identified as potential hosts of *Cryptosporidium*: *Salvelinus alpinus*, *Esox lucius*, *Coregonus lavaretus*, *Perca fluviatilis*, and *Rutilus rutilus*. The presence of Cryptosporidium spp. was found in 15 out of 41 fish (37%), distributed as follows: 13 (87%) *C*. *parvum*, 1 (7%) *C*. *molnari*, and 1 (7%) mixed infection (*C*. *parvum* and *C*. *molnari*). *C*. *molnari* was identified in the stomach, while *C*. *parvum* was found in the stomach and intestine. *C*. *molnari* was also detected in 1 out of 100 analyzed fillets. In order to identify *Cryptosporidium* subtypes, sequencing of the highly polymorphic 60-kDa glycoprotein (gp60) was performed. Among the *C*. *parvum* positive samples, three gp60 subtypes were identified: IIaA15G2R1, IIaA16G2R1, and IIaA17G2R1. Histological examination confirmed the presence of potential developmental stages of *C*. *parvum* within digestive epithelial cells. These observations suggest that *C*. *parvum* is infecting fish, rather than being passively carried. Since *C*. *parvum is a zoonotic* species, fish potentially contaminated by the same subtypes found in terrestrial mammals would be an additional source of infection for humans and animals, and may also contribute to the contamination of the environment with this parasite. Moreover, the risk of human transmission is strengthened by the observation of edible fillet contamination.

## Introduction

*Cryptosporidium*, a protozoan parasite that can cause severe diarrhea in a wide range of vertebrates including humans, is increasingly recognized as a parasite of a diverse range of wildlife species, including mammals, birds, reptiles, amphibians, and fish [1]. Although the epidemiology of cryptosporidiosis has been widely reported worldwide for different groups of animals, little biological, epidemiological and molecular data are available on *Cryptosporidium* infection in fish, even though the parasite has been already described and genetically characterized in more than 20 species of both freshwater and marine fish. *Cryptosporidium molnari*, the only currently recognized species infecting fish, was first identified in sea bream (*Sparus aurata*) and European sea bass (*Dicentrarchus labrax*) [2]. *Cryptosporidium scophthalmi* was detected in turbot (*Psetta maxima*, syn. *Scophthalmus maximus*) [3], but this species is still considered a *nomen nudum* due to a lack of genetic data [4].

*Cryptosporidium* species found in other groups of vertebrates have also been identified in fish, including *C*. *parvum*, *C*. *hominis*, *C*. *scrofarum* and *C*. *xiaoi*. Additionally, eight *Cryptosporidium* fish genotypes, and one *Cryptosporidium* rat III-like genotype, have been described in fish [4]. Recently, the species name *Cryptosporidium huwi* has been proposed for the piscine genotype 1 from the guppy (*Poecilia reticulata*) to reflect its genetic and biological differences from gastric and intestinal *Cryptosporidium* species [5].

In fish hosts, *Cryptosporidium* fish species and genotypes are located either in the stomach or intestine, as attested by histological analyses. Moreover, it has been reported that the parasite can cause clinical manifestations, such as emaciation, decrease in growth rate, anorexia, whitish feces, abdominal swelling, and ascites [2,3]. An increase in the mortality rate associated with *Cryptosporidium* infection has also been reported, particularly in larval and juvenile infected fish [6]. A significant correlation was found between the presence of the parasite and both fish weight and seasonality, the rate of infection being higher in fish weighing less than 100 grams and in the spring [7]. In addition, a relationship was observed between the presence of the parasite and the production stage in farmed fish [7].

It is notable that many results relating to fish *Cryptosporidium* infection were reported in farmed or aquarium fish [2,7,8]. However, little data are currently available regarding the molecular identification of *Cryptosporidium* species and genotypes in wild fish populations and, in particular, in edible fish. Indeed, only two studies have been conducted in Australia and Papua New Guinea on wild marine and freshwater fish [9,10].

**Fig 1:**
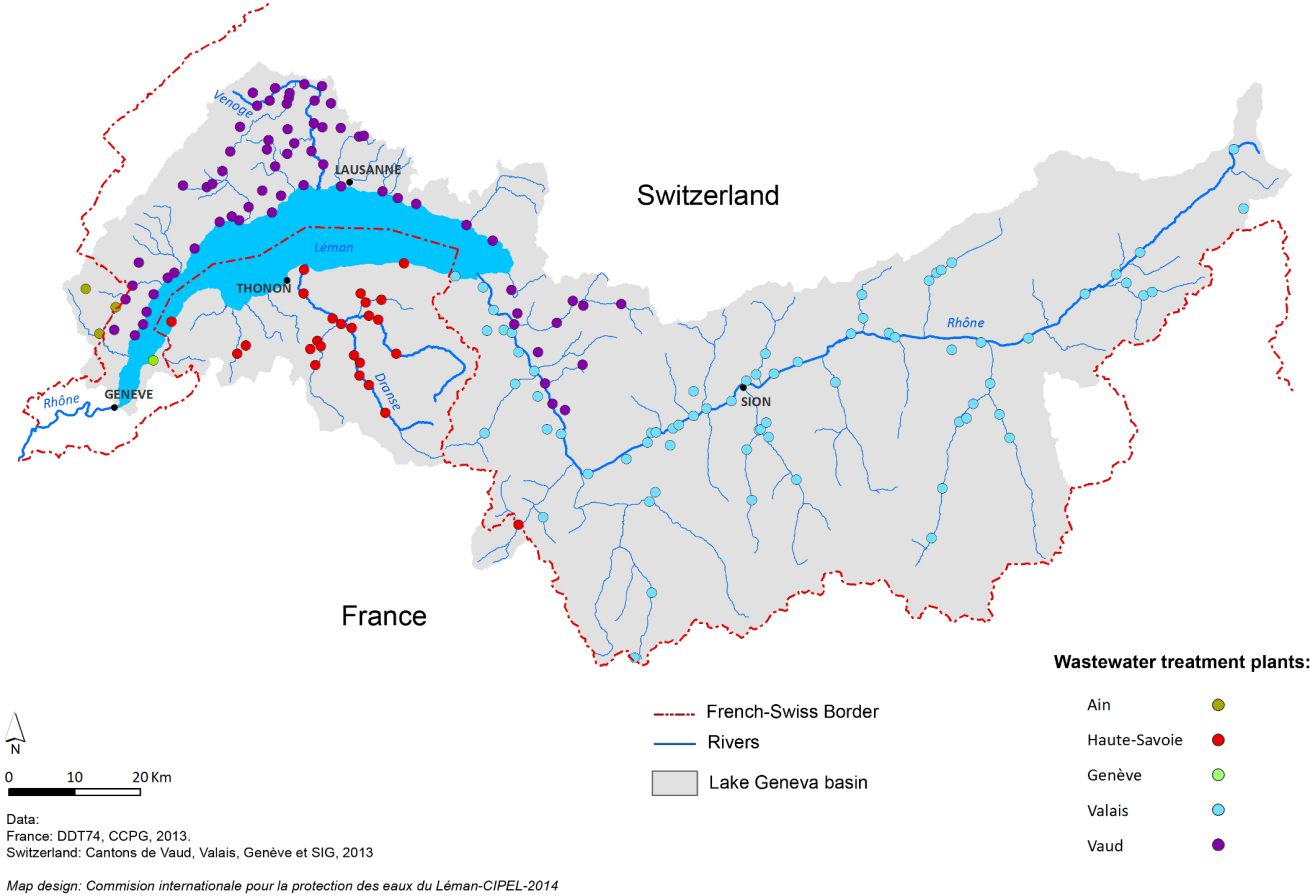
Map of the study area and sampling site (Thônon-les-Bains). Effluent from wastewater treatment plants discharged into the Lake Geneva catchment area (CIPEL: Commission Internationale pour la Protection des Eaux du Léman).

Therefore, the aim of our study was to evaluate the prevalence of *Cryptosporidium* species/ genotypes in freshwater edible fish hosts from Lake Geneva in France. Lake Geneva is located between Switzerland and France, and is the largest freshwater reservoir in Western Europe, with a surface area of 580 km^2^, a volume of 89 km^3^, and a maximum depth of 309 m (Fig 1). More than 1.5 million people (in France and Switzerland) live around this lake [11]. In addition, the local fish are quite often consumed as raw preparations by the local population at home or in restaurants located around the shores of the lake. Fish are also a source of income, as around 150 professional fishermen are registered as active on the lake.

## Materials and Methods

### Fish sampling

A total of 41 adult fish were purchased directly on the shores of the lake from local fishermen of Thônon-les-Bains (geographic coordinates: 46° 22' 0" North, 6° 29' 0" East), or Sechex, a small village located eight kilometers West of Thônon-les-Bains, in November 2011 (fall) and April 2013 (spring) (Fig 1). The weight, size, sex, origin and sexual maturity of each individual were determined (Table 1). For each fish, scratchings of the gastric and intestinal epithelia were performed after dissection, and the cells were preserved in the fixative RCL2 and stored at -20°C. A section of the stomach and bowel were also fixed in 10% buffered formalin. One hundred additional fillets (only cuts of fish flesh without viscera) of European perch (*Perca fluviatilis*) were purchased from the fishermen of Thônon-les-Bains, or St Gingolph (27 km Eeast of Thônon-les-Bains near the Swiss border, geographical coordinates: 46° 23' 0" North, 6° 40' 0" East) to evaluate potential contamination with *Cryptosporidium* spp. at this location. Slices of 2–3 mm were sampled and stored at—20°C in RCL2. No approval from Institutional Animal Care and Use Committee or ethics committee was necessary as no experiments that involved alive fish were performed. All fish examined were bought dead from professional fishermen, fishmongers and supermarkets selling fresh fish for consumption. Therefore, no sacrificial method was required. No fish sampled in this work was captured in a protected area and consequently, our sampling protocol did not need any specific permission for the location. Finally, no specimen included in the present work involved endangered or protected species.

**Table 1:** Freshwater fish specimens collected in Lake Geneva.

Fish code	Fish species	Common name	Size (cm)	Weight (g)	Sexual maturity	Sex	Presence of other parasites
5301	*Salvelinus alpinus*	Arctic char	40	610	ND	Male	Cestoda
5302	*Salvelinus alpinus*	Arctic char	37	504	ND	Male	Cestoda
5303	*Salvelinus alpinus*	Arctic char	37	530	ND	Male	Cestoda
5304	*Salvelinus alpinus*	Arctic char	38	548	ND	Male	Cestoda
5305	*Salvelinus alpinus*	Arctic char	29	224	ND	Male	Cestoda
5306	*Salvelinus alpinus*	Arctic char	38	580	ND	Male	Cestoda
5307	*Esox lucius*	Northern pike	37	328	ND	Female	Cestoda
5308	*Esox lucius*	Northern pike	35	328	No	Female	Cestoda
5309	*Lota lota*	Burbot	29	134	ND	Male	Cestoda
5310	*Lota lota*	Burbot	24	96	No	Female	Nematoda
5311	*Coregonus lavaretus*	European whitefish	31	276	No	Female	No
5312	*Coregonus lavaretus*	European whitefish	33	264	ND	Male	No
5313	*Coregonus lavaretus*	European whitefish	29	232	ND	Male	Cestoda
5314	*Coregonus lavaretus*	European whitefish	33	266	ND	Male	Cestoda
5315	*Coregonus lavaretus*	European whitefish	31	220	ND	Male	Cestoda
5316	*Coregonus lavaretus*	European whitefish	22	84	ND	ND	Cestoda
5317	*Perca fluviatilis*	European perch	11	16	ND	Male	Cestoda
5318	*Perca fluviatilis*	European perch	11	18	ND	Male	Cestoda
5319	*Perca fluviatilis*	European perch	11	16	ND	Male	Cestoda
5320	*Perca fluviatilis*	European perch	11	16	ND	ND	Cestoda
5321	*Perca fluviatilis*	European perch	42	1500	Yes	Female	Acantocephala
5322	*Perca fluviatilis*	European perch	29	318	No	Female	Trematoda digenea
5323	*Perca fluviatilis*	European perch	26	220	Yes	Female	Cestoda
5324	*Perca fluviatilis*	European perch	21	124	No	Female	Trematoda digenea
5325	*Rutilus rutilus*	Roach	18	42	No	Female	Cestoda
5326	*Lota lota*	Burbot	31	222	No	Female	No
5327	*Esox lucius*	Northern pike	53	1800	Yes	Female	Cestoda
5328	*Lota lota*	Burbot	25	97	ND	Male	Microsporidia
5329	*Coregonus lavaretus*	European whitefish	40	54	Yes	Female	Cestoda
5330	*Coregonus lavaretus*	European whitefish	33	54	ND	Male	Cestoda
5331	*Coregonus lavaretus*	European whitefish	36	54	ND	Male	Cestoda
5332	*Coregonus lavaretus*	European whitefish	40	54	Yes	Female	No
5333	*Coregonus lavaretus*	European whitefish	39	54	Yes	Female	No
5334	*Esox lucius*	Northern pike	69	2600	ND	Male	Monogenea
5335	*Squalius cephalus*	European chub	52	2300	Yes	Female	Monogenea
5336	*Esox lucius*	Northern pike	60	2000	Yes	Female	Monogenea
5337	*Perca fluviatilis*	European perch	27	30	Yes	Female	Acantocephala
5338	*Abramis brama*	Common bream	50	2000	ND	Male	*Trematoda digenea*
5339	*Perca fluviatilis*	European perch	15	45	Yes	Female	*Trematoda digenea*
5340	*Perca fluviatilis*	European perch	16	47	ND	Male	Cestoda
5341	*Perca fluviatilis*	European perch	16	56	Yes	Female	No

Fish from 5301 to 5326 were purchased from the fishermen of Thônon-les-Bains; Fish from 5327–5341 were purchased from the fishermen of Sechex

ND: Not determined.

### DNA extraction

Genomic DNA extraction was performed on 96-well plates, using the NucleoSpin Kit (Macherey-Nagel, GmbH & Co KG, Germany) according to the manufacturer’s protocol. The final DNA elution was 100 μl.

### Primer design and nested PCR

An alignment of the 18S rRNA gene sequences obtained from *Cryptosporidium* isolates characterized in fish (GenBank accession numbers: FJ769050, HM243547, HM243548, HM243549, HM243550, JF285332, JF285333, AY524773, HM989832, HM989833, HM989834, HM991857, GQ925452) [7] was performed using the BioEdit v7.0.1 package (http://www.mbio.ncsu.edu:BioEdit/bioedit.html). After identification of a target DNA fragment for nested 18S PCR common to all sequences, two sets of generate primers were selected within the hypervariable region. These degenerate primers were modified from those proposed by Ryan et al [12]. The external primer pair JerExtF (5’-GACATATCWTTYAAGTTTCTGACC-3’) (base pair position 292) and JerExtR (5’-CTGAAGGAGTAAGGAACAACC-3’) (base pair position 1007) amplified a DNA fragment of 784 bp. The internal primer pair JerIntF (5’-CCTATCAGCTTTMGACGGTAGG-3’) (base pair position 289) and JerIntR (5’-TCTAAGAATTTCACCTCTGACTG-3’) (base pair position 851) resulted in the amplification of a DNA fragment of 588 bp. For the first round of amplification, the PCR mixture contained 10 μl of DNA, 1x HotStarTaq Plus buffer, 2 mM MgCl_2_, 0.4 μM for each primer, 200μM dNTP each and 1.5U HotStarTaq Plus DNA polymerase (Qiagen Inc., Valencia, California) in a final volume of 50 μl. The PCR conditions were as follows: a denaturation step at 94°C for 10 min, followed by 40 cycles of 94°C for 45 sec, annealing at 67°C for 45 sec, and extension at 72°C for 1 min. The post-extension was completed at 72°C for 5 min. The second PCR amplification was performed in a 50 μl reaction volume containing 2 μl of the primary PCR product, 1xHotStarTaq Plus buffer, 3 mM MgCl_2_, 0.4 μM for each primer, 200 μM dNTP each and 1.5 U HotStarTaq Plus DNA polymerase. The PCR conditions were identical to those in the first round. Nested 18S PCR reactions were conducted using a PTC 200 thermocycler (MJ Research, Waltham, USA). The PCR products were analyzed on a 2% agarose gel and visualized by ethidium bromide staining.

### DNA sequencing and analysis

To identify *Cryptosporidium* species at the molecular level, positive nested 18S PCR products were purified and sequenced directly on both strands, using the forward and reverse primers from the second round, by the company Genoscreen (Institut Pasteur de Lille, France). The sequences obtained were aligned using the BioEdit v7.0.1 package, and then compared with the sequences of *Cryptosporidium* published on the NCBI server (http://www.ncbi.nlm.nih.gov/BLAST/) using the basic local alignment search tool (BLAST) program. Isolates genotyped as *C*. *parvum* were further subtyped using a second nested PCR that amplifies a fragment of the 60 kDa glycoprotein (gp60) gene, as described [13]. The amplified DNA fragments were purified, sequenced, and analyzed as described above.

### Histological analysis

The stomach and intestine of the fish were removed, fixed in 10% buffered formalin, and paraffin-embedded specimens were sectioned to a thickness of 5 μm to be processed using standard staining techniques (Hematoxylin & Eosin). Inflammation in digestive sections was scored as follows: 0, no inflammation; +1, moderate inflammation, focally distributed; +2, moderate inflammation, widely distributed; +3, severe inflammation, widely distributed throughout the section. The sections were examined by a pathologist using a Leica DMRB microscope equipped with a Leica digital camera connected to an Imaging Research MCID analysis system (MCID Software, Cambridge, UK).

### Nucleotide sequence accession numbers

The 18S rRNA nucleotide sequences obtained in this study were deposited in the GenBank database under the accession numbers KP939333-KP939354.

## Results

The molecular analysis of digestive tissues identified the presence of *Cryptosporidium* spp. in 15 out of 41 fish, representing a frequency of 37%. The fish species Arctic char (*Salvelinus alpinus*) (4/6), Northern pike (*Esox lucius*) (2/5), European whitefish (*Coregonus lavaretus*) (4/11), European perch (*Perca fluviatilis*) (4/12), and roach (*Rutilus rutilus*) (1/1) were identified as potential new hosts for *Cryptosporidium* spp. (Table 2).

**Table 2:** *Cryptosporidium* species and subtypes in wild freshwater fish from Lake Geneva identified at the 18S rDNA and GP60 loci.

Code	Fish species	Fish common name	Organ	*Cryptosporidium* species 18S	Percentage of identity with reference sequence *	SNP** position	SNP**	GP60
5302	*Salvelinus alpinus*	Arctic char	Intestine	*C*. *parvum*	99.8%	347	T/C	NA
5303	*Salvelinus alpinus*	Arctic char	Intestine	*C*. *parvum*	99.6%	347	T/C	IIaA17G2R1
						435	C/T	
5304	*Salvelinus alpinus*	Arctic char	Stomach	*C*. *parvum*	99.8%	390	G/A	IIaA15G2R1
			Intestine	*C*. *parvum*	99.8%	145	A/G	IIaA15G2R1
5305	*Salvelinus alpinus*	Arctic char	Intestine	*C*. *parvum*	99.6%	300	T/C	IIaA15G2R1
						507	A/G	
5307	*Esox lucius*	Northern pike	Stomach	*C*. *molnari*	98.3%	314	A/T	NA
						322	T/A	
						324	T/C	
						329	C/T	
						341	A/G	
						370	A/T	
						376	A/T	
						377	C/T	
						506	G/A	
			Intestine	*C*. *parvum*	99.4%	244	G/A	IIaA17G2R1
						347	T/C	
						496	T/C	
5308	*Esox lucius*	Northern pike	Stomach	*C*. *molnari*	98.3%	314	A/T	NA
						322	T/A	
						324	T/C	
						329	C/T	
						341	A/G	
						370	A/T	
						376	A/T	
						377	C/T	
						506	G/A	
5311	*Coregonus lavaretus*	European whitefish	Stomach	*C*. *parvum*	100%	-	-	IIaA15G2R1
			Intestine	*C*. *parvum*	99.8%	437	T/C	IIaA17G2R1
5312	*Coregonus lavaretus*	European whitefish	Stomach	*C*. *parvum*	99.6%	324	T/C	IIaA17G2R1
						475	T/C	
	*Coregonus lavaretus*	European whitefish	Intestine	*C*. *parvum*	99.2%	87	A/G	-
						151	A/G	
						390	G/A	
						491	T/C	
5314	*Coregonus lavaretus*	European whitefish	Stomach	*C*. *parvum*	99.8%	235	A/G	-
5316	*Coregonus lavaretus*	European whitefish	Stomach	*C*. *parvum*	99.8%	390	G/A	-
5318	*Perca fluviatilis*	European perch	Stomach	*C*. *parvum*	99.6%	300	T/C	IIaA15G2R1
						507	A/G	
5320	*Perca fluviatilis*	European perch	Stomach	*C*. *parvum*	99.8%	211	C/T	-
5322	*Perca fluviatilis*	European perch	Stomach	*C*. *parvum*	99.8%	27	G/A	IIaA16G2R1
			Intestine	*C*. *parvum*	100%	-	-	IIaA16G2R1
5323	*Perca fluviatilis*	European perch	Stomach	*C*. *parvum*	100%	-	-	IIaA15G2R1
5325	*Rutilus rutilus*	Roach	Stomach	*C*. *parvum*	100%	-	-	IIaA17G2R1
	* *		Intestine	*C*. *parvum*	100%	-	-	-

* The reference sequences for *C*. *parvum* and *C*. *molnari* are: KJ939305 and HM243550, respectively.

**SNP: Single nucleotide polymorphism

NA: Not available.

The sequence analysis of the 18S rDNA locus identified two species of *Cryptosporidium*, distributed as follows: 13 *C*. *parvum* (87%), 1 *C*. *molnari* (7%), and 1 mixed infection (*C*. *molnari* and *C*. *parvum*) (7%). In 9 of the 15 infected fish, the presence of *Cryptosporidium* spp. was found either in the stomach or intestine, while in the 6 remaining infected fish, *Cryptosporidium* spp. were present in both organs. The selective extraction of DNA from these organs, followed by nested 18S PCR and sequencing, confirmed the presence of *C*. *molnari* only in the stomach of fish, while *C*. *parvum* was found in both stomach and intestine. Among the stomach samples, two were positive for *C*. *molnari*, and 10 were positive for *C*. *parvum*. Among the intestinal samples, eight were positive for *C*. *parvum* only. The 18S rRNA gene sequences of 5 out of 19 isolates of *C*. *parvum* found either in the stomach or intestine were 100% identical to that of a previously described species of *C*. *parvum* (GenBank: KJ939305 [7]), while 14 isolates exhibited single nucleotide polymorphisms (SNPs). It is common to identify sequence differences and variations such as single nucleotide polymorphisms (SNPs) that can be associated to genetic diversity according to the degree of homology. SNPs were distributed as follows: only 1 SNP for 8 isolates, 2 SNPs for 4 isolates, 3 SNPs for 1 isolate, and 4 SNPs for one isolate (Table 2). All SNPs identified in the *C*. *parvum* isolates corresponded to transition mutations. The two isolates identified as *C*. *molnari* were identical but showed 9 SNPs in comparison to the *C*. *molnari* reference sequence (GenBank: HM243550[7] (Table 2). In particular, 5 SNPs were associated with transition mutations, and 4 SNPs (in positions 322, 330, 378, 384) were associated with transversion mutations between adenine and thymine (A/T). The SNPs could not be associated with a specific sampling site (gastric vs. intestinal site) or with a specific fish species.

In order to identify *Cryptosporidium* subtypes, sequencing of the highly polymorphic 60-kDa glycoprotein (gp60) was performed. Partial sequences of the gp60 gene were subsequently obtained for 13 isolates identified as *C*. *parvum*. Three different subtypes were identified as follows: IIaA15G2R1 (6/13), IIaA17G2R1 (5/13), and IIaA16G2R1 (2/13) (Table 2).

**Fig 2:**
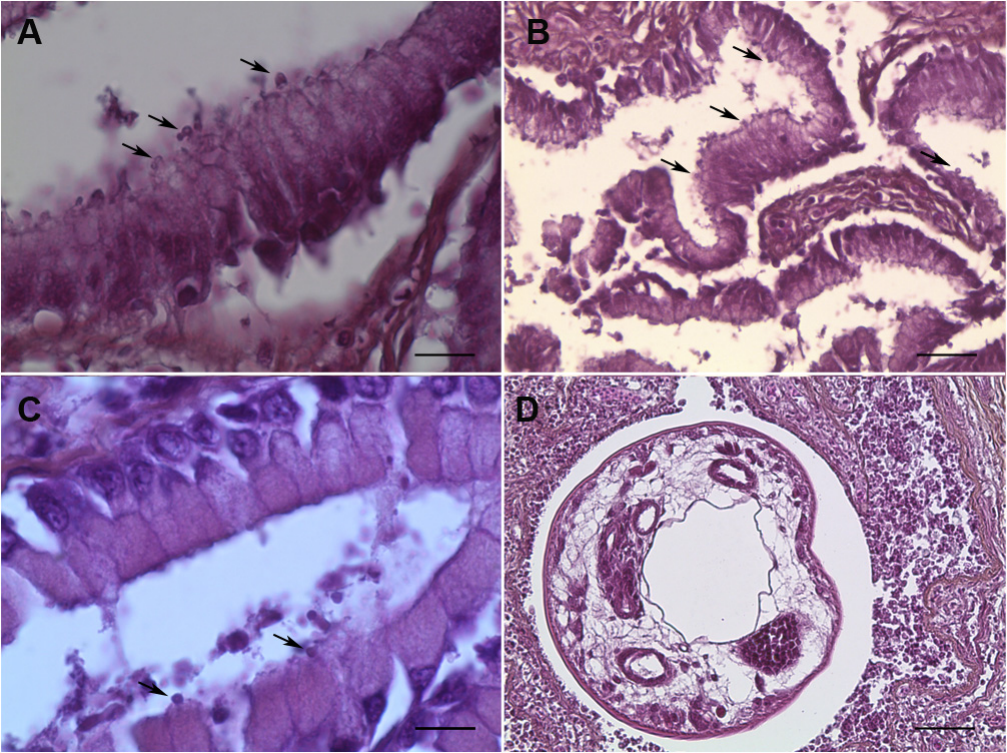
Stained sections of the digestive tract of fish. A. Presence of round bodies suggestive of the developmental stages of *C*. *parvum* was observed in the apical position (arrows) within the epithelial cells of gastric glands. Bar = 25 μm. B. Presence of round bodies suggestive of the developmental stages of *C*. *parvum* observed in the apical position (arrows) within the intestinal epithelial cells. Bar = 75 μm. C. Presence of round bodies suggestive of the developmental stages of *C*. *molnari* observed in the apical position (arrows) within the epithelial cells of gastric glands. Bar = 25 μm. D. Section of a nematode in the intestinal mucosa, surrounded by a severe inflammatory reaction. Bar = 200 μm. Hematoxylin & Eosin staining.

Following histological examination of sections either from the stomach or intestine, the presence of *Cryptosporidium*-like bodies within the cells of the digestive epithelium was confirmed in samples from 10 *C*. *parvum*-positive fish (Fig 2A, 2B and 2C; Table 3). An inflammatory reaction with leukocyte infiltration was observed occasionally. The presence of other intestinal parasites, identified as nematodes, was confirmed in histological sections of two fish (Fig 2D, Table 2). The histological analysis of the remaining fish was not possible due to autolysis of tissues.

**Table 3:** Histological examination of digestive organs of different fish species from Lake Geneva infected by *Cryptosporidium* spp.

Fish code	Fish species	Fish common name	Organ	Histological examination*	*Cryptosporidium species (18S rDNA)*
5302	*Salvelinus alpinus*	Arctic char	Intestine	Inflammation: +1	*C*. *parvum*
				Intracellular *Cryptosporidium*-like bodies	
5303	*Salvelinus alpinus*	Arctic char	Intestine	ND	*C*. *parvum*
5304	*Salvelinus alpinus*	Arctic char	Stomach	Inflammation: 0 Intracellular	*C*. *parvum*
				*Cryptosporidium*-like bodies	
			Intestine	ND	*C*. *parvum*
5305	*Salvelinus alpinus*	Arctic char	Intestine	Inflammation: +1	*C*. *parvum*
				Intracellular *Cryptosporidium*-like bodies	
5307	*Esox lucius*	Northern pike	Stomach	ND	*C*. *molnari*
			Intestine	ND	*C*. *parvum*
5308	*Esox lucius*	Northern pike	Stomach	Zones of autolysis	*C*. *molnari*
				Inflammation: 0 Intracellular *Cryptosporidium*-like bodies	
5311	*Coregonus lavaretus*	European whitefish	Stomach	ND	*C*. *parvum*
			Intestine	Zones of autolysis	*C*. *parvum*
				Inflammation: 0 Intracellular *Cryptosporidium*-like bodies	
5312	*Coregonus lavaretus*	European whitefish	Stomach	Zones of autolysis	*C*. *parvum*
				Inflammation: 0 Intracellular *Cryptosporidium*-like bodies	
			Intestine	Zones of autolysis	*C*. *parvum*
				Inflammation: 0 Intracellular *Cryptosporidium*-like bodies	
5314	*Coregonus lavaretus*	European whitefish	Stomach	Inflammation: 0 Intracellular *Cryptosporidium*-like bodies	*C*. *parvum*
5316	*Coregonus lavaretus*	European whitefish	Stomach	Inflammation: 0 Intracellular *Cryptosporidium*-like bodies	*C*. *parvum*
5318	*Perca fluviatilis*	European perch	Stomach	Inflammation: +3	*C*. *parvum*
				Presence of a nematode	
5320	*Perca fluviatilis*	European perch	Stomach	Autolysis	*C*. *parvum*
5322	*Perca fluviatilis*	European perch	Stomach	ND	*C*. *parvum*
			Intestine	Inflammation: +3	*C*. *parvum*
				Presence of a nematode	
5323	*Perca fluviatilis*	European perch	Stomach	Inflammation: 0 Intracellular *Cryptosporidium*-like bodies	*C*. *parvum*
5325	*Rutilus rutilus*	Roach	Stomach	Inflammation: 0 Intracellular *Cryptosporidium*-like bodies	*C*. *parvum*
	* *		Intestine	Inflammation: 0 Intracellular *Cryptosporidium*-like bodies	*C*. *parvum*

ND: Not done

* Inflammation in digestive sections was scored as follows: 0, no inflammation; +1, moderate inflammation, focally distributed; +2, moderate inflammation, widely distributed; +3, severe inflammation, widely distributed throughout the section.

The potential contamination of fish flesh with *Cryptosporidium* spp. was evaluated. One hundred fish fillets of European perch (*Perca fluviatilis*) were analyzed by nested PCR and sequencing. The presence of *C*. *molnari* was detected in only one fillet. The 18S rRNA gene sequence of this *C*. *molnari* isolate was identical to that of the two isolates found in the stomach of two Northern pike (Table 2).

## Discussion

This study reports the first epidemiological and molecular data on the presence of *Cryptosporidium* in fish in France. The overall frequency of *Cryptosporidium* spp. in fish sampled from Lake Geneva was high, reaching 37%. Previous studies have reported a high prevalence of *Cryptosporidium* spp. in fish, but mainly in juvenile marine fish. For instance, Sitjà-Bobadilla et al. reported 100% *C*. *scophthalmi* prevalence in juvenile turbot in Europe [7]. In contrast, a recent study in Australia found no *Cryptosporidium* isolates in freshwater fish [9], while a *Cryptosporidium* prevalence of 0.2% was found in wild freshwater species in Papua New Guinea [10]. Therefore, even if the comparative data is scarce, this is to our knowledge the first time that *Cryptosporidium* has been detected at a very high prevalence in freshwater fish.

Five new species of fish hosts for *Cryptosporidium* were identified: Arctic char (*Salvelinus alpinus)*, Northern pike *(Esox lucius)*, European whitefish *(Coregonus lavaretus)*, European perch *(Perca fluviatilis)* and Roach (*Rutilus rutilus)*. Although it is generally accepted that the prevalence of *Cryptosporidium* is higher in juvenile fish, all of the fish analyzed in our study were adults, according to size and weight, and according to sexual maturity when this parameter could be determined (Table 1).

Two species of *Cryptosporidium* were detected in fish hosts: *C*. *molnari* and *C*. *parvum*. *C*. *molnari* was identified in freshwater aquaculture fish [14], but this is the first time that this parasite species has been found in wild freshwater fish. The 18S rRNA gene sequences of the 3 *C*. *molnari* isolates identified in our study were 98% identical to those of the *C*. *molnari* reference sequences collected from the databases. Interestingly, these three sequences amplified from different individuals presented the same points of mutation, suggesting the circulation of the same parasite isolates in the lake environment.

A matter of importance to public health was the high rate of detection of *C*. *parvum* among fish hosts, as this species is the most common source of zoonotic infections [4]. Previous studies in Papua New Guinea and Australia also reported consistent detection of *C*. *parvum* in fish [9,10]. We speculate that the presence of *C*. *parvum*, and in particular the IIa subtype, in fish samples from Lake Geneva could be due to waterborne contamination with human and animal waste. In fact, the zoonotic *C*. *parvum* IIa subtype family has predominantly been found in calves and humans in North America, Europe, and Australia [15,16]. In addition, even if we did not search for the presence of *Cryptosporidium* in the lake water, it is well known that *Cryptosporidium* oocysts are found in groundwater, lakes, rivers, estuaries, and oceans, as a consequence of the great amount of feces from humans, pets, and domesticated or wild animals that is discharged, dumped, or carried in runoff into these waters [17]. In particular, in Lake Geneva, an increase in fecal bacteria of human and animal origin was described in sediment contaminated with wastewater treatment plant effluent, suggesting the presence of both human and animal sources of fecal pollution in the lake environment [11]. In parallel, it has been suggested that when fecal bacteria is present in water, *Cryptosporidium* could be present as well, and even though water quality monitoring and water treatment can reduce the presence of pathogens, they do not ensure absolute safety, due to the fact that *Cryptosporidium* oocysts are highly resistant [18].

Partial sequences of the gp60 gene subsequently amplified from *C*. *parvum* isolates allowed the identification of three different subtypes belonging to the IIa family, as follows: IIaA15G2R1, IIaA16G2R1, and IIaA17G2R1. The IIaA15G2R1 subtype has also been identified consistently in Papua New Guinea in mackerel scad, *Decapterus maracellus* [10], a wild marine fish. The subtypes IIaA15G2R1 and IIaA17G2R1 have been identified in cattle [19], the first of which is the most dominant zoonotic *Cryptosporidium* subtype infecting dairy cattle and humans in industrialized countries [4]. Indeed, the IIaA15G2R1 subtype represents up to 75% of the identified *Cryptosporidium* subpopulation in French calves [16]. On the other hand, the IIaA16G2R1 subtype has been identified in diarrheic calves [20–22] and also in wild boars (*Sus scrofa*) [23]. In rural areas, it is well known that animals can cohabit with livestock, often by sharing grazing and water sources. Other zoonotic *Cryptosporidium* species already identified in marine fish such as *C*. *hominis C*. *xiaoi* and *C*. *scrofarum* were not found in this study [4].

Since *C*. *parvum* is a zoonotic species, fish potentially contaminated by the same subtypes infecting terrestrial mammals would be an additional source of infection for humans and other animals, and may also contribute to the contamination of the environment with this parasite. However, it is not clear if fish are only carriers of *C*. *parvum*, or if *C*. *parvum* can develop its life cycle and multiply in this fish host.

In order to clarify this question, histological analysis of digestive tissues from *C*. *parvum*-positive fish was performed, and round bodies suggestive of *C*. *parvum* developmental stages were observed in an apical position within the cells, either in the stomach or intestine. These observations suggest that *C*. *parvum* is actually infecting fish, rather than being passively carried. Fluorescent-antibody staining assay using an anti-*Cryptosporidium* antibody (Crypto Cel immunofluorescence test, Cellabs, Brookvale, New South Wales, Australia) was tried to confirm the detection of *Cryptosporidium* oocysts from fish tissues but unfortunately, no signal was detected. This failure was probably due to the vulnerability of the oocyst antigens to formalin as was already described, particularly after more than one month of formalin fixation which was the case in our study [14]. Mild to moderate inflammation was occasionally found in gastrointestinal tissues, but we could not determine whether it was *Cryptosporidium* that was causing this reaction, since co-infection with other parasites was present. In some cases, the histological analysis of fish was not possible due to autolysis of tissues.

Furthermore, to evaluate a potential contamination of fish fillets with *Cryptosporidium* spp., 100 fish fillets of European perch (*Perca fluviatilis)* were analyzed by nested 18S PCR and sequencing, and the presence of *C*. *molnari* was detected in fillets from one individual. Fillet contamination with *C*. *molnari* could occur as a consequence of evisceration of the infected fish during the cleaning and preparation process. Although previous studies have shown no conclusive evidence of transmission of fish-hosted *Cryptosporidium* to mammals [24], the presence of the parasite also in fillets clearly highlights the risk of *Cryptosporidium* infection to humans, either when handling fish or consuming raw or undercooked fish carrying zoonotic species of *Cryptosporidium*. In our study, only *C*. *molnari*, apparently a non-pathogenic species for humans, was isolated from perch fillets. However, *C*. *parvum* isolated from the fish digestive tract could certainly be present in fish fillets. Further studies should be done to clarify this aspect.

One study in Maryland consistently reported that urban anglers are at a risk of contracting cryptosporidiosis from exposure received while fishing and consuming caught fish with a mean probability of infection of almost one [25]. Another study showed that blue crabs can transfer *C*. *parvum* oocysts to people who handle the crustaceans [26]. In addition, it has been reported that immunosuppressed patients are at risk of contracting cryptosporidiosis, either by contact with fish during preparation and handling, or by consumption of undercooked fish [27].

It was not unexpected to find *Cryptosporidium* in fish from Lake Geneva, as this parasite has already been found to be responsible for a human outbreak occurring in 2003 due to the contamination of the water supply network, in the nearby city of Divonne-les-Bains, affecting more than 700 individuals [28]. In addition, in Switzerland, a study reported the presence of *C*. *parvum* in samples collected from the drinking water distribution system in alpine rural regions, and it was suspected that the drinking water was contaminated by grazing cattle [29]. However, future studies should be conducted to detect the presence of the parasite in the lake environment.

In conclusion, these findings suggest that the transmission of *Cryptosporidium* could potentially occur in the interfaces between human, livestock, and fish populations. In fact, the wide host range of *Cryptosporidium* spp., together with the high output of oocyst shedding, allows a high level of contamination of the environment [23]. In particular, for fish hosts, the dispersion and transmission of zoonotic parasites would be facilitated by the aquatic habitat of the host that could potentially release fully sporulated oocysts contributing to the perpetuation of *Cryptosporidium* circulation. Finally, fish may be a good sentinel for the detection of water contamination caused by sewage or agricultural runoff.
